# Sustained attention in adult ADHD: time-on-task effects of various measures of attention

**DOI:** 10.1007/s00702-015-1426-0

**Published:** 2015-07-24

**Authors:** Lara Tucha, Anselm B. M. Fuermaier, Janneke Koerts, Rieka Buggenthin, Steffen Aschenbrenner, Matthias Weisbrod, Johannes Thome, Klaus W. Lange, Oliver Tucha

**Affiliations:** 1 Department of Clinical and Developmental Neuropsychology, University of Groningen, Grote Kruisstraat 2/1, 9712 TS Groningen, The Netherlands; 2 Department of Clinical Psychology and Neuropsychology, SRH Clinic Karlsbad-Langensteinbach, Karlsbad-Langensteinbach, Germany; 3 Department of Psychiatry and Psychotherapy, SRH Clinic Karlsbad-Langensteinbach, Karlsbad-Langensteinbach, Germany; 4 Section for Experimental Psychopathology, Centre for Psychosocial Medicine, University of Heidelberg, Heidelberg, Germany; 5 Department of Psychiatry and Psychotherapy, University of Rostock, Rostock, Germany; 6 Department of Experimental Psychology, University of Regensburg, Regensburg, Germany

**Keywords:** Adult ADHD, Cognition, Neuropsychological assessment, Attention, Sustained attention, Time-on-task effects

## Abstract

Neuropsychological research on adults with ADHD showed deficits in various aspects of attention. However, the majority of studies failed to explore the change of performance over time, so-called time-on-task effects. As a consequence, little is known about sustained attention performance of adults with ADHD. The aim of the present study was therefore to test the hypothesis of sustained attention deficits of adults with ADHD. Twenty-nine adults with ADHD and 30 healthy individuals were assessed on four 20-min tests of sustained attention, measuring alertness, selective attention, divided attention and flexibility. The deterioration of performance over time (time-on-task effects) was compared between patients with ADHD and healthy individuals to conclude on sustained attention performance. Compared to healthy individuals, patients with ADHD showed significant deficits of medium size in selective attention and divided attention. Furthermore, medium sustained attention deficits was observed in measures of alertness, selective attention and divided attention. This study supports the notion of sustained attention deficits of adults with ADHD.

## Introduction

Symptoms of inattention, such as distractibility and concentration difficulty, are core features of attention deficit hyperactivity disorder (ADHD) (American Psychiatric Association 2013). Whereas ADHD had been historically understood as an exclusive childhood disorder, longitudinal and follow-up studies of children with ADHD suggested that core symptoms of ADHD persist into adulthood in about 30–60 % of cases (Biederman et al. 1998; Mannuzza and Klein 2000). In the last decade, a large body of research has been performed to characterize cognitive impairments of adults with ADHD. It has been revealed that adults with ADHD show difficulties in a variety of functions related to attention, including working memory, inhibition, selective attention, divided attention and flexibility (Dinn et al. 2011; Fuermaier et al. 2013b; Rohlf et al. 2012; Schoechlin and Engel 2005; Tucha et al. 2006a, 2008). The majority of these studies evaluated cognitive functions of adults with ADHD in comparison to a control group or normative data by the application of neuropsychological tests over short time periods (i.e., 3–5 min).

Sustained attention, in contrast, can be distinguished from these various aspects of attention, as sustained attention requires participants to maintain the focus to one or more sources of information over a relatively long and unbroken period of time (Van Zomeren and Brouwer 1994). Maintaining attention is crucial for successful daily functioning, affecting both the private setting (e.g., driving a vehicle, contributing to conversations) as well as the occupational setting (e.g., working on a computer). The neuropsychological assessment of sustained attention, therefore, requires participants to remain focused and ready to react to the presentation of target stimuli over a longer period of time (i.e., 15–20 min). As the demand to remain focused over a longer period of time is crucial in this context, the actual performance of sustained attention can be assessed by examining the *change* of performance *over time*, as it is shown by so-called time-on-task (TOT) effects (van der Meere and Sergeant 1988a). Consequently, a deficit in sustained attention can only be inferred if the deterioration of performance over time (TOT effects) exceeds the natural decline of attention performance over time (group-by-time interaction) (Tucha et al. 2009; van der Meere and Sergeant 1988a).

Considering that deficits of sustained attention are part of the diagnostic criteria for ADHD as defined in the DSM (American Psychiatric Association 2013) and that such deficits can be very detrimental for daily life functioning of individuals, it is not surprising that a substantial number of studies have been performed to examine sustained attention in adults with ADHD (Avisar and Shalev 2011; Epstein et al. 1998, 2001; Gansler et al. 1998; Johnson et al. 2001; Marchetta et al. 2008; Riccio and Reynolds 2001; Seidman et al. 1998; Tucha et al. 2009). However, these studies were associated with several conceptual and methodological problems limiting the conclusions drawn from these studies and their results. For example, sustained attention has primarily been assessed by using vigilance tests (e.g., variants of the Continuous Performance Test (CPT) (Epstein et al. 1998; Huang-Pollock et al. 2012)). Vigilance tests require participants to remain attentive to infrequently occurring stimuli under very monotonous conditions. Daily life situations requiring sustained attention, however, usually demand higher activation levels, frequent interactions with the environment and flexible switching between tasks (e.g., when driving a vehicle: tracking and monitoring changing locations of neighboring vehicles and reacting appropriately to them by adapting the speed of the own vehicle). Based on face validity of task characteristics, it can be argued that sustained attention as it is required in daily life may be better assessed by tests of maintained alertness, selective attention, divided attention or flexibility. Moreover, the majority of studies on sustained attention of adults with ADHD calculated summary scores over the total duration of the test and by this failed to report changes of performance over time (Avisar and Shalev 2011; Gansler et al. 1998; Riccio and Reynolds 2001; Seidman et al. 1998), while only a few studies examined the decline of performance over time (TOT effects) (Epstein et al. 1998, 2001, Johnson et al. 2001; Marchetta et al. 2008; Tucha et al. 2009). As no information was presented concerning changes of test performance over time in the majority of studies, sustained attention deficits cannot be concluded from these data. Those studies considering changes of performance over time in their analyses included several types of variables such as speed of responses, speed variability of responses, omission errors as well as commission errors. The results of these analyses largely advocated an undisturbed level of sustained attention of adults with ADHD in these paradigms by failing to demonstrate a greater decline of performance over time as compared to control participants (absence of group-by-time interaction) (Epstein et al. 1998, 2001; Tucha et al. 2009). Marchetta et al. (2008), however, found evidence for a greater decline of performance over time (presence of group-by-time interaction) in a measure of speed variability of a CPT, suggesting a sustained attention deficit of adults with ADHD.

In conclusion, based on paradigms and analyses techniques used in previous studies, there is only little evidence that adults with ADHD exhibit deficits of sustained attention. Sustained attention has primarily been assessed with vigilance tasks so far which may certainly be useful to measure a selective aspect of sustained attention, but may fail to measure a variety of other aspects of attention as required in daily life, i.e., the prolonged application of selective and divided attention as well as sustained alertness and flexibility. This is in particular surprising since previous research demonstrated (Tucha et al. 2006b, 2008) that both children and adults with ADHD suffer from deficits in these components of attention when being assessed for shorter periods (3–5 min). The aim of the present study was, therefore, to test the hypothesis of sustained attention deficits of adults with ADHD by applying tests measuring maintained alertness, selective attention, divided attention and flexibility. Time-on-task effects of various measures of attention were compared between a group of healthy individuals and a group of adults with ADHD in order to analyze sustained attention deficits.

## Methods

### Participants

#### Patients with ADHD

Twenty-nine adults with ADHD participated in the study. Patients were self-referred or referred from local psychiatrists or neurologists to the Department of Psychiatry and Psychotherapy, SRH Group, Karlsbad-Langensteinbach, Germany. A diagnostic assessment for ADHD in adulthood as well as participation in the research project was offered to all participants. Diagnostic assessments were performed by experienced clinicians associated to the Department of Psychiatry and Psychotherapy and involved a clinical psychiatric interview according to DSM-IV criteria for ADHD as devised by Barkley and Murphey (Barkley and Murphy 1998) including the retrospective diagnosis of ADHD in childhood (DSM-IV criteria) and current symptoms. All diagnoses were made on mutual agreement between two clinicians. Moreover, all participants completed two standardized self-report rating scales designed to quantify current and retrospective ADHD symptoms (Rösler et al. 2008). In the diagnostic assessment of the 29 patients with ADHD, eight patients met DSM-IV criteria for ADHD—predominantly inattentive type (ADHD-I) and 21 patients met criteria for ADHD—combined type (ADHD-C) (none of the patients met criteria for ADHD—hyperactive-impulsive type (ADHD-H)). Based on information obtained from an anamnestic interview and medical files, nine of the 29 patients with ADHD were diagnosed with one or more comorbid disorders, including mood disorders (*n* = 5), anxiety disorders (*n* = 3), posttraumatic stress disorders (*n* = 2), eating disorders (*n* = 2), and obsessive–compulsive disorder (n = 1). Three patients with ADHD reported to have been treated with stimulant medication in the past; however, none of the participants was treated with stimulant medication at the time of the study. Moreover, two patients were treated with antidepressant medication for a prolonged period of time because of affective disorders. Further, none of the participants reported having a history of substance abuse disorder during the previous 6 months and none reported a history of neurological disorder including head injury. Characteristics of patients with ADHD are presented in Table 1.

**Table 1:** Characteristics and neuropsychological functions of healthy individuals (control) and patients with ADHD (ADHD)

Characteristics	Control (*n* = 30)	ADHD (*n* = 29)	*t* (57)	*p*	ES (*d*)^a^
Age (in years)	33.4 ± 12.5	33.5 ± 11.1	0.04	0.970	0.01
Gender (female/male)	16/14	15/14			0.03
Intellectual functions (IQ)	106.6 ± 14.1	101.5 ± 13.5	1.34	0.168	0.37
School education (in years)	12.4 ± 1.3	11.1 ± 1.9	2.94	0.005*	0.80
WURS-K^c^	7.9 ± 6.3	43.8 ± 14.7	12.25	<0.001*	3.19
ADHD self-report	7.7 ± 4.8	34.8 ± 9.0	14.59	<0.001*	3.78

Neuropsychological functions as measured in the first time block (5-min testing)^c^	Control (*n* = 30)	ADHD (*n* = 29)	*Z*	*p*	ES (*r*)^b^
Alertness					
Reaction time (ms)	252 ± 41	293 ± 89	1.918	0.055	0.25
Variability of reaction time	126 ± 13	133 ± 22	1.435	0.151	0.19
Number of omissions	0.17 ± 0.46	1.28 ± 3.93	1.435	0.072	0.23
Selective attention					
Reaction time (ms)	356 ± 69	420 ± 75	1.435	0.004*	0.37
Variability of reaction time	123 ± 8	126 ± 8	1.268	0.205	0.17
Number of omissions	0.17 ± 0.46	1.10 ± 2.30	1.903	0.057	0.25
Divided attention					
Reaction time (ms)	515 ± 202	544 ± 160	1.198	0.231	0.16
Variability of reaction time	139 ± 12	143 ± 13	1.009	0.313	0.13
Number of omissions	2.07 ± 2.08	5.07 ± 4.25	3.336	0.001*	0.43
Flexibility					
Speed costs	0.23 ± 0.19	0.29 ± 0.26	0.918	0.359	0.12
Accuracy costs	1.40 ± 1.85	2.04 ± 3.96	0.479	0.632	0.06

* Significant at *p* < 0.05

^a^Effect size is indicated by Cohen’s *d*

^b^Effect size is indicated by Cohen’s *r*

^c^Wender Utah Rating Scale—short version

#### Healthy individuals

Furthermore, 30 healthy individuals were assessed. Healthy participants were recruited from the local community via public announcements, word-of-mouth and through contacts of the researchers involved. None of the healthy individuals reported having a history of neurological or psychiatric diseases and none were taking any medication known to affect the central nervous system at the day of the assessment. All healthy individuals completed the same self-rated questionnaires for current and retrospective ADHD symptoms prior to the assessment (Rösler et al. 2008). Scores of all healthy participants were below the cutoff value suggesting a clinical level of ADHD symptom severity. Intellectual functions of all participants were measured using the Multiple Choice Vocabulary Test (Lehrl 1995). Patients and healthy individuals did not differ in age, intellectual functions (Table 1) and gender (*χ* ^2^(1) = 0.015; *p* = 0.902). However, healthy individuals had a significantly higher school education than patients with ADHD. As expected, healthy individuals scored lower on both questionnaires for ADHD symptoms.

### Measures

#### Self-report scales for ADHD symptoms

Two standardized self-report rating scales designed to quantify ADHD symptoms currently and retrospectively were applied to all participants (Rösler et al. 2008). Childhood ADHD symptoms were self-rated with the short version of the Wender Utah Rating Scale (WURS-K) including 25 items on a five-point scale (Ward et al. 1993). Severity of current ADHD symptoms was self-rated with the ADHD self-report scale (Rösler et al. 2008) consisting of 18 items on a four-point scale corresponding to the diagnostic criteria of DSM-IV (American Psychiatric Association 1994; Rösler et al. 2008). A sum score was calculated for each rating scale.

#### Intellectual functions

Intellectual functions (IQ) were measured using the Multiple Choice Vocabulary Test (Lehrl 1995). This test consists of 37 lines, each comprising of one authentic word and four fictitious words. The participants were required to find the authentic word by underlining it. The Multiple Choice Vocabulary Test is a valid and short test procedure which provides a measure for intellectual functioning (Lehrl et al. 1995).

#### Assessment of sustained attention

Sustained attention was assessed with the Vienna Test System (VTS) (Schuhfried 2013), a computerized test battery for the measurement of various neuropsychological functions. Four tests of the VTS were adapted for the present study, all of which had originally been developed under theoretical based considerations to assess different dimensions of attention, i.e., alertness, selective attention, divided attention and flexibility (Gmehlin et al. 2012; Häusler and Sturm 2009; Sturm 2006; Van Zomeren and Brouwer 1994). Alertness, selective attention and divided attention were measured using adaptations of tests for perception and attention functions (WAF), i.e., WAFA, WAFS and WAFG (Sturm 2006). Studies on the construct validity of these tests supported the theoretical based model of different dimensions of attention (Häusler and Sturm 2009). Flexibility was measured with an adaption of the SWITCH (Gmehlin et al. 2012), a recently published test for the measurement of cognitive flexibility (task switching). All tests were adapted (prolonged) with regard to the test duration so that each test took about 20 min. For each test, an instruction phase and a short practice phase preceded the actual test phase. The practice phase was repeated if necessary until participants understood the task instructions adequately (more than 80 % correct responses during the practice phase). The total duration of the test phase of each test was about 20 min and was split into 4 time blocks consisting each of the same number of target stimuli (each time block took about 5 min).

*Alertness* Measures for intrinsic alertness reflect the response readiness without any external preparatory cue (intensity aspect of attention). In the present study, maintained intrinsic alertness was measured with an adaptation of the test for perception and attention functions called ‘alertness’ (WAFA—subtest intrinsic visual) (Schuhfried 2013; Sturm 2006). The reliability of the original test version was reported to be 0.93. In this test, participants were instructed to fixate on a cross in the center of a computer screen and to press a button on a response panel as soon as a black dot (target stimulus) appeared in the center of the screen. Each target stimulus was presented for 1500 ms but disappeared as soon as a response was given. If participants failed to respond within these 1500 ms, an omission error was registered. A total number of 340 target stimuli were presented, whereas the time between the presentations of two subsequent target stimuli (inter-stimulus interval (ISI)) varied between 3000 and 5000 ms. The mean reaction time of responses (in ms), the mean variability of reaction times (standard deviation of reaction times in ms) and the number of omissions were calculated for each time block.

*Selective attention* Measures for selective attention reflect the ability to focus attention on particular features of a task but to suppress reactions to irrelevant features. In the present study, maintained selective attention was measured with an adaption of the test for perception and attention functions called ‘selective attention’ (WAFS—subtest unimodal visual) (Schuhfried 2013, Sturm 2006). The reliability of the original test version was reported to be 0.95. In this test, a series of stimuli (circles, squares or triangles) was presented in consecutive order in the center of a computer screen. Each stimulus was presented for 1500 ms. After 500 ms of each stimulus presentation, a change may take place, i.e., the stimulus may get lighter or darker or stays the same. The participants were requested to react as quickly as possible to changes in circles and squares but to ignore changes in triangles. A response was given by pressing a button on a response panel. A total number of 475 stimuli were presented in pseudorandomized order of which 100 stimuli required a response. An omission error was counted, if no response was given during the presentation of a target stimulus (1000 ms presentation time of each target stimulus, i.e., between 500 and 1500 ms after stimulus onset). The time between the presentations of two subsequent stimuli (inter-stimulus interval (ISI)) was 1000 ms. The mean reaction time of responses (in ms), the mean variability of reaction times (standard deviation of reaction times in ms) and the number of omissions were calculated for each time block.

*Divided attention* Measures for divided attention reflect the ability to divide attention between a number of information channels. In the present study, maintained divided attention was measured with an adaption of the test for perception and attention functions called ‘divided attention’ (WAFG—subtest crossmodal visual auditory) (Schuhfried 2013; Sturm 2006). The reliability of the original test version was reported to be 0.97. In this test, participants were required to monitor simultaneously one visual and one auditory stimulus channel. In the visual stimulus channel, a series of 400 stimuli were presented in consecutive order in the center of a computer screen. Each stimulus consisted of a pair of shapes (two circles, two rectangles or one of both), one displayed upon the other. Each stimulus was presented for 1500 ms. After 500 ms of each stimulus presentation, a change may take place in one or both shapes of the stimulus presented, i.e., the shape may get lighter or stays the same. The participants were requested to react as quickly as possible if the same kind of shape (circle or rectangle) became lighter twice in succession (in two subsequent stimuli). The time between the presentations of two subsequent stimuli (inter-stimulus interval (ISI)) was 1000 ms. In the auditory stimulus channel, a series of 400 sounds, each of the same pitch, were presented in consecutive order to participants. Each sound was presented for 1500 ms. After 500 ms of each sound presentation, a change may take place, i.e., the tone may get softer or stays the same. The participants were requested to react as quickly as possible if the sound became softer twice in succession (in two subsequent sounds). The time between the presentations of two subsequent sounds (inter-stimulus interval (ISI)) was 1000 ms. The task (visual and auditory information channel) requested 100 responses in total, each by pressing the same specified button on a response panel. An omission error was counted, if no response was given during the presentation of a target stimulus (1000 ms presentation time of each target stimulus, i.e., between 500 and 1500 ms after stimulus onset). The presentation order of stimuli in both information channels was pseudorandomized. The mean reaction time of responses (in ms), the mean variability of reaction times (standard deviation of reaction times in ms) and the number of omissions were calculated for each time block.

*Flexibility* Measures for cognitive flexibility reflect the ability to switch flexibly between different tasks. In the present study, maintained cognitive flexibility was measured with an adaptation of the SWITCH (Gmehlin et al. 2012; Schuhfried 2013). The reliability of the original version of the SWITCH varies between 0.81 and 0.98 for the different measures of the test. In this test, a series of stimuli was presented in consecutive order in the center of a computer screen. Stimuli differed with regard to *shape* (circle or triangle) and *color* (black or grey). Participants were instructed to respond as quickly as possible either to *shape* or *color* of the stimulus presented. Responses were given by pressing one of two predefined buttons on a response panel (buttons were symmetrically placed on the left and right side of the panel’s middle axis). When a response to the *shape* of the stimulus was requested (independent from the color), a triangle required a response on the left button, whereas a circle required a response on the right button. When a response to the *color* of the stimulus was requested (independent from the shape), a grey stimulus required a response on the left button whereas a black stimulus required a response on the right button. Each stimulus was presented for a maximum of 5000 ms but disappeared as soon as a response was given (one trial). The time between a response in the present trial and the presentation of the stimulus in the subsequent trial [response–stimulus interval (RSI)] was 750 ms. Participants were instructed to alternately switch their focus between *shape* and *color* in every other trial. That means participants were requested to respond to the *shape* in two consecutive trials, subsequently respond to the *color* in two consecutive trials, switch back to the *shape* for another two trials, etc. (i.e., shape–shape–color–color–shape–shape–color–color, etc.). In case of incorrect responses of any type, participants were corrected by the test and prompted to give the correct response. This was achieved by a message automatically appearing on the screen referring to the incorrect response made in the respective trial and indicating the correct response the participant was supposed to give. The test continued as soon as the participant gave the prompted correct response. In total, 560 stimuli were presented throughout the task in pseudorandomized presentation order. Two variables were calculated as depended variables indicating switching costs, i.e., *speed costs* and *accuracy costs*. For the calculation of *speed costs*, the mean reaction time was calculated for the correct trials requiring responses to the *other type of stimulus* (shape or color) as the preceding trial (mean reaction time for switching trials). In addition, the mean reaction time was calculated for the correct trials requiring responses to the *same type of stimulus* (shape or color) as the preceding trial (mean reaction time for repetitive trials). The calculation of reaction times was based on the trials with correct responses, while trials with incorrect or omitted responses were not taken into account. The variable *speed costs* were calculated by subtracting the mean reaction time for switching trials from the mean reaction time for repetitive trials. Accordingly, for the calculation of *accuracy costs*, the percentage of the correct trials was calculated that required responses to the *same type of stimulus* (shape or color) as the preceding trial (percentage of correct repetitive trials). In addition, the percentage of correct trials was calculated that required responses to the *other type of stimulus* (shape or color) as the preceding trial (percentage of correct switching trials). The variable *accuracy costs* were calculated by subtracting the percentage of correct repetitive trials from the percentage of correct switching trials. *Speed costs* and *accuracy costs* were calculated for each time block.

### Procedure

All participants were invited to take part in the study on a voluntary basis and received no reward for participation. Before the start of the assessment, all participants were informed about the aim of the study and it was pointed out that all data collected in the research project will be analyzed anonymously and will not affect clinical assessment and treatment. It was also emphasized that participants had the right to withdraw from the study at any time. At the beginning of the experiment, descriptive and anamnestic information (e.g., age, school education, medical history) was obtained. Subsequently, four tests of sustained attention were performed, each taking about 20 min. Participants were free to use either their right or left hand to perform the tests on the VTS. A break followed the execution of each test. Short breaks (1–2 min) were allowed between the first test and the second test as well as between the third and the fourth test. However, a longer break (10–15 min) was taken between the second and the third test. The order of the four tests (alertness, selective attention, divided attention and flexibility) was counterbalanced across participants. A complete counterbalanced design resulted in 24 different test orders which were each allocated to patients and healthy controls. Remaining participants were each randomly allocated to one of the 24 test orders. The total duration of the assessment was about 120 min.

### Ethics statement

The study was conducted in compliance with the Helsinki Declaration. Ethical approval was obtained by the ethics committee of the medical faculty of the University of Heidelberg, Germany. All participants gave written informed consent prior to the assessment and were debriefed at the end of the assessment.

### Statistical analysis

Nonparametric statistical tests were performed to analyze the data since an assumption check for parametric statistics showed considerable violations, such as non-normal distributions of most measures (as indicated by Kolmogorov–Smirnov tests). Attention performance during the first time block of each test was compared between healthy individuals and patients with ADHD by applying Mann–Whitney *U* tests for independent samples. These analyses were performed in order to explore group differences in attention within short test periods which place only limited requirements on sustained attention. Mann–Whitney *U* tests were also applied to compare attention performance of participants in the last time block of each test in order to compare group differences in attention after a prolonged period of testing. Moreover, performance of sustained attention was analyzed (TOT effects). For this purpose, attention performance between the first time block and the last time block was compared separately for healthy individuals and individuals with ADHD by applying Wilcoxon signed-rank test for dependent samples. Furthermore, ipsative scores were calculated for each individual and each measure of attention by subtracting attention performance of the last time block from the attention performance of the first time block. Ipsative scores were then compared between healthy individuals and individuals with ADHD by applying Mann–Whitney *U* tests. An alpha level was set to 0.05 for all tests. Furthermore, effect sizes were calculated for all comparisons. Whereas the significance criterion represents the standard measure for analyzing whether a phenomenon exists, the effect size refers to the magnitude or the importance of effects (Cohen 1988; Glass et al. 1981; Pedhazur and Pedhazur Schmelkin 1991). For pairwise comparisons, negligible effects (*r* < 0.1), small effects (0.1 ≤ *r* < 0.3), medium effects (0.3 ≤ *r* < 0.5) and large effects (*r* ≥ 0.5) were distinguished (Cohen 1988). Statistical significances in the present analysis must be interpreted with caution as multiple testing may lead to alpha-error accumulation. Effect sizes indicate the magnitude of effects independently from statistical significance and are thus not affected by alpha-error accumulation. Interpretations of findings of the present study are thus largely based on effect sizes.

Furthermore, explorative analyses were carried out in order to examine the association between TOT effects of various measures of attention. This was done by correlating (Spearman rank correlation) ipsative scores with each other for a collapsed group of healthy individuals and individuals with ADHD. According to Cohen (1988), negligible effects (*r* < 0.1), small effects (0.1 ≤ *r* < 0.3), medium effects (0.3 ≤ *r* < 0.5) and large effects (*r* ≥ 0.5) were distinguished.

## Results

### Attention performance in first time block

Group comparisons between healthy individuals and individuals with ADHD revealed a significantly decreased performance of patients with ADHD in reaction times of the selective attention task as well as in the number of omissions of the divided attention task (Table 1). The calculation of effect sizes confirmed that adults with ADHD performed considerably poorer than healthy subjects, as indicated by medium differences in selective attention (number of omissions) and divided attention (reaction time). The remaining differences were of small size with the exception of a negligible difference between groups in the accuracy costs of the flexibility task. Table 1 presents significances and effect sizes for univariate group comparisons.

### Sustained attention performance

#### Alertness

**Fig. 1:**
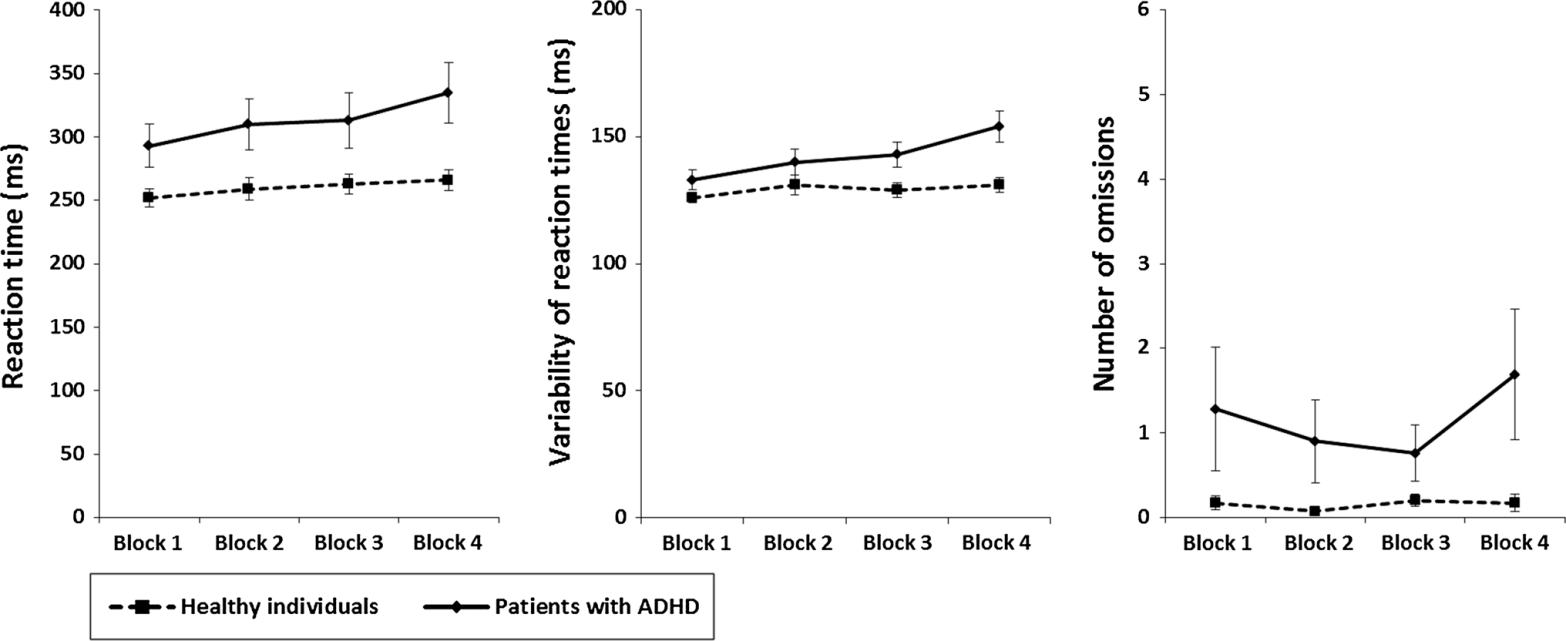
Sustained alertness. Mean reaction time (*left panel*), mean variability of reaction times (*middle panel*), and number of omission errors (*right panel*) of both samples of participants for each of the four 5-min time blocks (mean and standard error) of the alertness task. *Error bars* indicate standard errors

With regard to *reaction times* in the alertness task, a significant group difference of small size was found for the last time block (*Z* = 2.161, *p* = 0.031, *r* = 0.28). Both healthy individuals (*Z* = −2.303, *p* = 0.021, *r* = 0.30) as well patients with ADHD (*Z* = −3.103, *p* = 0.002, r = 0.40) displayed a significant and medium decline of performance over time (TOT effects); however, the groups did not differ significantly in this decline and the difference was only small (*Z* = −1.744, *p* = 0.081, *r* = 0.23) (Fig. 1). With regard to *variability of reaction times*, a medium and significant group difference was found in the last time block (*Z* = −3.656, *p* < 0.001, *r* = 0.48). While patients with ADHD showed a significant and large deterioration of performance over time (TOT effects) (*Z* = −4.002, *p* < 0.001, *r* = 0.52), such an effect was not observed in healthy individuals as indicated by a non-significant and small effect size (*Z* = −0.878, *p* = 0.380, *r* = 0.11). Consequently, a comparison of TOT effects demonstrated a significantly greater decrease of performance over time in patients with ADHD than in healthy individuals (*Z* = −3.254, *p* = 0.001, *r* = 0.42). The effect was of medium size (Fig. 1). The analysis of *number of* *omissions* in the alertness task revealed a significant difference between groups of medium size in the last time block (*Z* = −2.933, *p* = 0.003, *r* = 0.38). However, neither healthy individuals (*Z* = −0.087, *p* = 0.931, *r* = 0.01) nor patients with ADHD (*Z* = −1.061, *p* = 0.288, *r* = 0.14) showed a significant change of performance over time (TOT effects), as noted by negligible to small effect sizes. Consequently, only a non-significant small difference was observed between groups with respect to their deterioration of performance over time (TOT effects) (*Z* = −1.206, *p* = 0.228, *r* = 0.16) (Fig. 1).

#### Selective attention

**Fig. 2:**
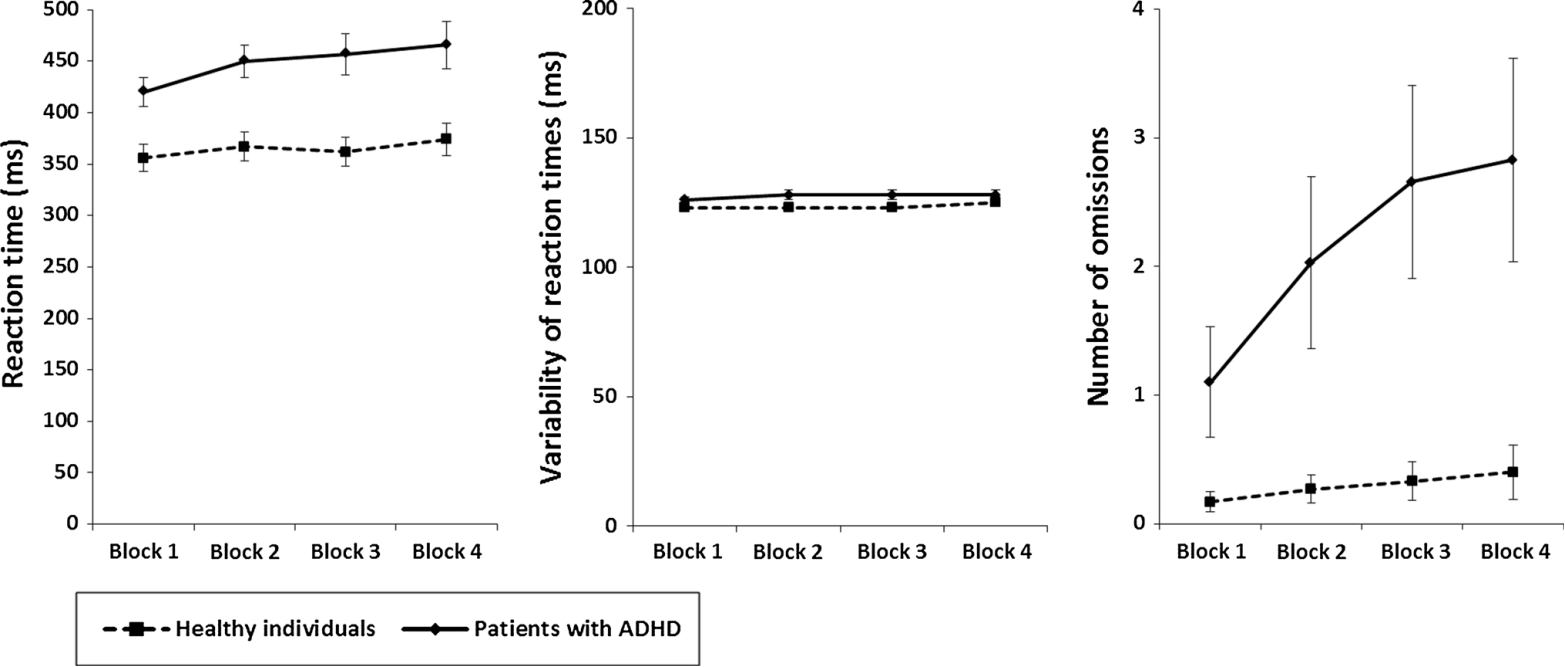
Sustained selective attention. Mean reaction time (*left panel*), mean variability of reaction times (*middle panel*), and mean number of omission errors (*right panel*) of both samples of participants for each of the four 5-min time blocks of the selective attention task. *Error bars* indicate standard errors

The analysis of *reaction times* in the selective attention test revealed a significant and medium difference between groups in the last time block (*Z* = −3.033, *p* = 0.002, *r* = 0.39). Whereas healthy individuals did not change significantly in reaction times between the first and the last time block (*Z* = −1.275, *p* = 0.202, *r* = 0.17), patients with ADHD showed a significant increase in reaction times over time (*Z* = −2.952, *p* = 0.003, *r* = 0.38) (TOT effects). Effects were of small and medium size, respectively. The increase in reaction times, however, did not differ considerably (non-significant small difference) between groups (*Z* = −1.607, *p* = 0.108, *r* = 0.21) (Fig. 2). Analyzing the *variability of reaction times* revealed a non-significant and small difference between groups in the last time block (*Z* = −1.017; – = 0.309, *r* = 0.13). Neither healthy individuals (*Z* = −1.378, *p* = 0.165, *r* = 0.18) nor patients with ADHD (*Z* = −1.148, *p* = 0.251, *r* = 0.15) showed a significant change of performance over time, resulting in a non-significant difference of TOT effects between groups (*Z* = −0.175, *p* = 0.861, *r* = 0.02). Effects were of negligible to small size (Fig. 2). With regard to the *number of omissions*, a significant and medium difference between groups was found in the last time block (*Z* = −3.530, *p* < 0.001, *r* = 0.46). Whereas a non-significant and small change of performance over time was observed for healthy individuals (*Z* = −1.098, *p* = 0.272, *r* = 0.14), patients with ADHD showed a significant and medium decline of performance over time (*Z* = −3.553, *p* < 0.001, *r* = 0.46). Significant and medium differences in TOT effects were found between healthy individuals and patients with ADHD (*Z* = −3.343, *p* = 0.001, *r* = 0.44) (Fig. 2).

#### Divided attention

**Fig. 3:**
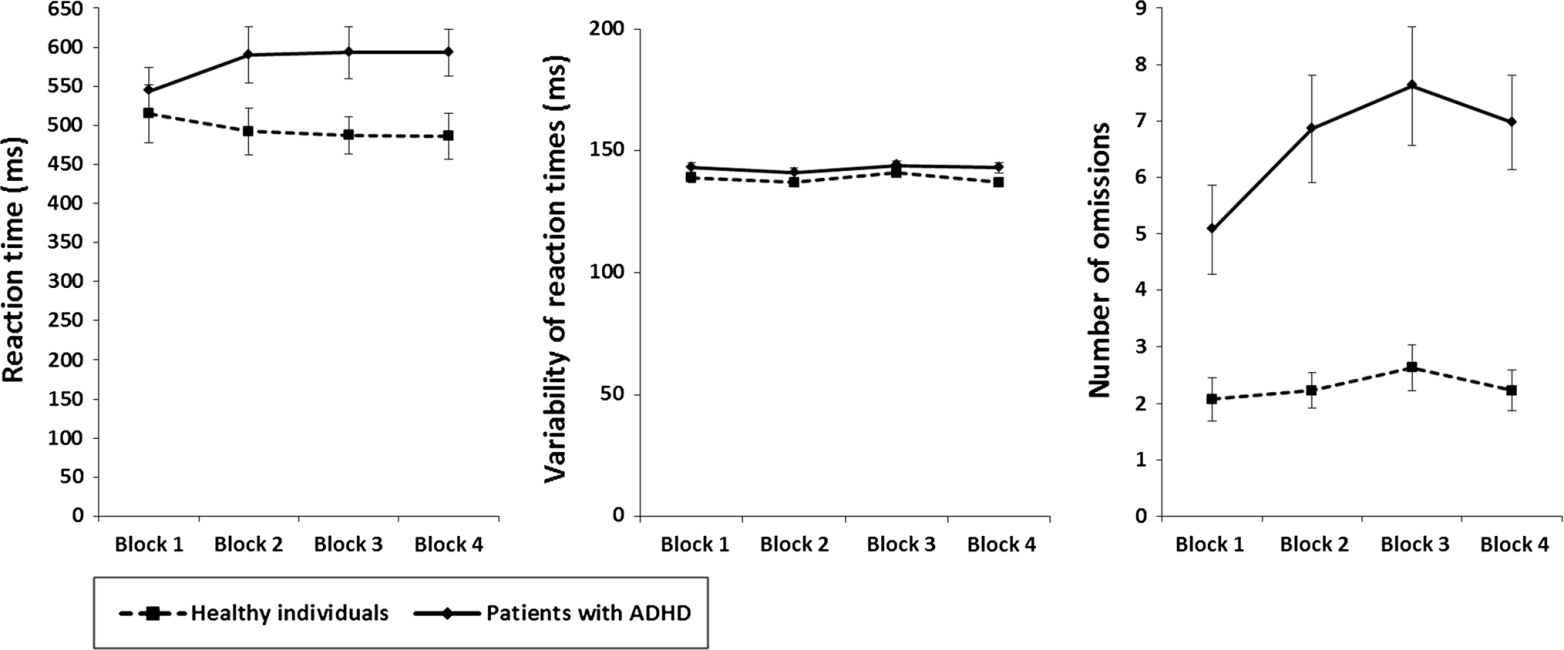
Sustained divided attention. Mean reaction time (*left panel*), mean variability of reaction times (*middle panel*), and mean number of omission errors (*right panel*) of both samples of participants for each of the four 5-min time blocks of the divided attention task. *Error bars* indicate standard errors

Statistical analysis of *reaction times* in the divided attention task indicated a significant difference of medium size between healthy individuals and patients with ADHD in the last time block (*Z* = −3.033, *p* = 0.002, *r* = 0.39). Whereas healthy individuals showed a small and non-significant TOT effects in reaction times (*Z* = −1.111, *p* = 0.267, *r* = 0.15), patients with ADHD displayed a significant and medium decline in performance over time (*Z* = −2.876, *p* = 0.004, *r* = 0.37). The difference between groups in the decline of reaction times was significant and of medium size (*Z* = −2.843, *p* = 0.004, *r* = 0.37) (Fig. 3). Analyzing the *variability of reaction times* for the test of divided attention revealed a non-significant difference of small size between groups in the last time block (*Z* = −1.768, *p* = 0.077, *r* = 0.23). Neither healthy individuals (*Z* = -0.963, p = 0.336, r = 0.13) nor patients with ADHD (*Z* = −0.171, *p* = 0.864, *r* = 0.02) demonstrated significant TOT effects. Effects were of negligible to small effects. A non-significant difference of small size was also found when comparing TOT effects between groups (*Z* = −0.766, *p* = 0.444, *r* = 0.10) (Fig. 3). With regard to the *number of omissions* made occurring in the last time block, a significant difference of large size was found between groups (*Z* = −4.293, *p* < 0.001, *r* = 0.56). Whereas healthy individuals exhibited non-significant and negligible TOT effects (*Z* = −0.210, *p* = 0.833, *r* = 0.03), patients with ADHD showed a significant and medium deterioration of performance over time (*Z* = −2.604, *p* = 0.009, *r* = 0.34). The TOT effects observed in patients with ADHD were significantly greater than TOT effects of healthy individuals (*Z* = −2.207, *p* = 0.027, *r* = 0.29). The effect was of small size (Fig. 3).

#### Flexibility

**Fig. 4:**
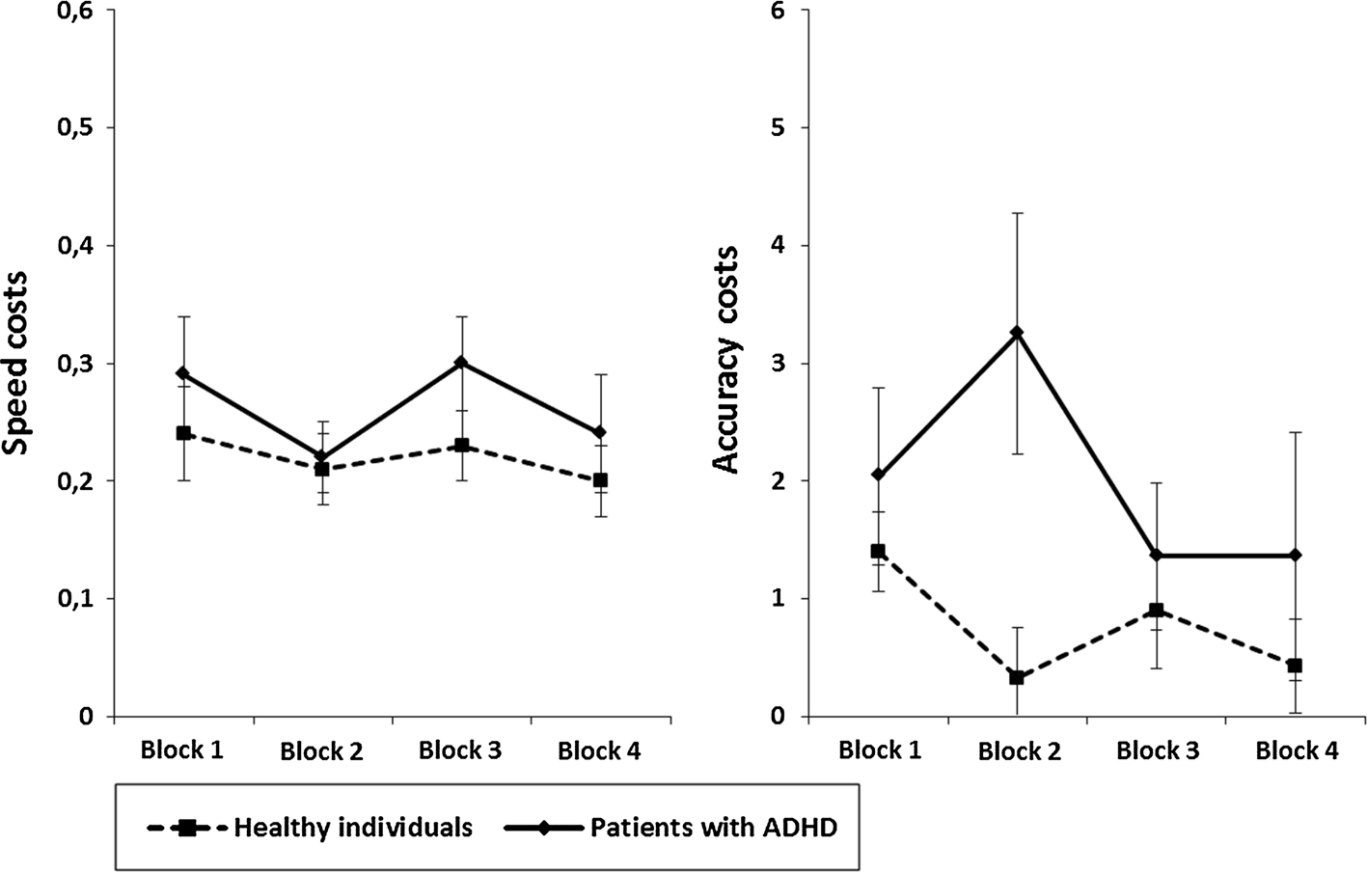
Sustained flexibility. Mean speed costs (*left panel*) and mean accuracy costs (*right panel*) of both samples of participants for each of the four 5-min time blocks of the flexibility task. *Error bars* indicate standard errors

Flexibility was assessed by measures of speed costs and accuracy costs. A comparison of *speed costs* between groups revealed a non-significant effect of negligible size in the last time block (*Z* = −0.202; *p* = 0.840, *r* = 0.03). Neither healthy individuals (*Z* = −0.977, *p* = 0.329, *r* = 0.13) nor patients with ADHD (*Z* = −1.025, *p* = 0.305, *r* = 0.13) showed significant TOT effects. Effects were of small size. A non-significant negligible effect was also observed when comparing TOT effects between groups (*Z* = −0.265, *p* = 0.791, *r* = 0.03) (Fig. 4). Analysis of *accuracy costs* showed a non-significant negligible difference between groups in the last time block (*Z* = −0.455, *p* = 0.649, *r* = 0.06). Again, neither healthy individuals (*Z* = −1.694, *p* = 0.090, *r* = 0.22) nor individuals with ADHD (*Z* = −0.993, *p* = 0.321, *r* = 0.13) showed significant TOT effects. Effects were of small size. A comparison of TOT effects between groups yielded a non-significant negligible effect (*Z* = −0.063, *p* = 0.950, *r* = 0.01) (Fig. 4).

#### Association between measures of sustained attention

Explorative correlation analysis was performed to examine the association between the various measures of sustained attention. Ipsative scores indicating TOT effects of various measures of attention were correlated with each other using Spearman rank correlation coefficients. Healthy individuals and patients with ADHD were collapsed for the purpose of this analysis to obtain a single sample of sufficient size and a wide range of scores. Correlation coefficients and significances are presented in Table 2. Correlations ranged from negligible to medium size. Significant correlations were found between five pairs of measures, i.e., between measures of the divided attention task (omissions and reaction times: *r* = 0.357; *p* = 0.006), measures of the selective attention task (reaction times and omissions: *r* = 0.339; *p* = 0.009), between omissions occurring during the divided attention task and omissions in the selective attention task (*r* = 0.380; *p* = 0.003), between the standard deviation of reaction times in the alertness task and reaction times in the divided attention task (*r* = 0.343; *p* = 0.008), as well as between the accuracy costs in the flexibility task and omissions in the alertness task (*r* = −0.336; *p* = 0.010) (Table 2).

**Table 2:** Spearman rank correlations (rho) between TOT effects of various measures of attention for a collapsed group of healthy individuals and patients with ADHD (*n* = 59)

rho	Al-RT	Al-SD	Al-Om	Sel-RT	Sel-SD	Sel-Om	Div-RT	Div-SD	Div-Om	Flex-Acc
Al-SD	−0.059									
Al-Om	0.106	0.032								
Sel-RT	0.178	0.020	−0.057							
Sel-SD	0.048	−0.027	−0.076	−0.021						
Sel-Om	0.233	0.209	0.152	0.339**	−0.121					
Div-RT	0.066	0.343**	−0.008	0.171	0.010	0.218				
Div-SD	−0.225	0.058	0.233	−0.065	0.078	−0.100	0.054			
Div-Om	0.162	0.081	0.146	0.190	0.186	0.380**	0.357**	0.153		
Flex-Acc	−0.057	−0.143	−0.336*	0.128	−0.025	0.017	−0.016	0.046	0.151	
Flex-Speed	−0.215	0.002	−0.167	0.022	−0.035	−0.121	0.057	0.195	0.124	0.219

*Al*-*RT* alertness-reaction times, *Al-SD* alertness-standard deviation of reaction times, *Al-Om* alertness-omissions, *Sel-RT* selective attention-reaction times, *Sel-SD* selective attention-standard deviation of reaction times, *Sel-Om* selective attention-omissions, *Div-RT* divided attention-reaction times, *Div-SD* divided attention-standard deviation of reaction times, *Div-Om* divided attention-omissions, *Flex-Acc* flexibility-accuracy costs, *Flex-Speed* flexibility-speed costs

* Significant at *p* < 0.05

** Significant at *p* < 0.01

## Discussion

The present study examined the hypothesis of sustained attention deficits of adults with ADHD. Initial data analysis of attention performance in the first time block (5-min testing) demonstrated medium deficits of adults with ADHD in selective attention (increased reaction times) and divided attention (increased number of omissions). These results are in accordance with a large body of previous research which found deficits of adults with ADHD in various aspects of attention, including selective and divided attention (Fuermaier et al. 2013a; Hervey et al. 2004; Schoechlin and Engel 2005; Tucha et al. 2006a, 2008). However, deficits in flexibility of adults with ADHD could not be obtained in the present study. This appears surprising in the light of robust findings of previous research suggesting executive dysfunctions as the primary cognitive deficit in both children and adults with ADHD (Schoechlin and Engel 2005; Willcutt et al. 2005). With regard to cognitive flexibility, several studies found deficits of adults with ADHD in measures of set-shifting and task switching (Halleland et al. 2012; Rohlf et al. 2012; Tucha et al. 2005), which were linked in neuroimaging studies to reduced activation in bilateral inferior frontal cortices (Cubillo et al. 2010). However, even though executive dysfunctions play a prominent role in cognitive theories on ADHD, meta-analyses demonstrated that deficits in measures of cognitive flexibility were small and inconsistent across studies, suggesting that set-shifting may be a poor candidate for a primary neuropsychological deficit in ADHD (Hervey et al. 2004; Willcutt et al. 2005). Furthermore, the non-significant differences between groups of the present study may have resulted from a high variability of task performance between participants.

For the purpose of this study, particular emphasis was given to the effects of time on task. An analysis of TOT effects revealed that patients with ADHD exhibited a significant deterioration of performance over time in several measures of attention, including tests of alertness, selective attention and divided attention. Effect sizes of TOT effects ranged from small to large size, indicating that prolonged execution of neuropsychological tasks is cognitively exhausting, resulting in decreased task performance with regard to speed, variability of speed, and accuracy with ongoing task duration. The deterioration of patients’ performance over time is underlined by significant differences of medium to large size between healthy individuals and patients with ADHD in most measures of attention during the last time block (last block of 5-min testing).

With respect to sustained attention, patients with ADHD showed a greater decline of performance over time as compared to healthy individuals (group differences of TOT effects). This was shown by mainly medium effects in several functions, i.e., alertness (variability of reaction times), selective attention (number of omissions) as well as divided attention (reaction times and number of omissions). Analysis of reaction times in the test of divided attention, however (see Fig. 3), reveals an increase in reaction times of patients with ADHD from the first time block to the second time block, whereas rather constant mean reaction times were observed in the second, third and fourth time block. The reaction times (denoting response readiness and arousal) might therefore not indicate a primary deficit of sustained divided attention, since a constant increase in reaction times with ongoing task duration was not observed. However, there was an increase in the number of omissions over time (indicating a careless response style or distractibility) which was also observed at subsequent time blocks (i.e., second to third time block), indicating a deficit of sustained divided attention. Taking together, the present findings suggest that patients with ADHD suffer from deficits in sustained attention, i.e., in sustained alertness, sustained selective attention and sustained divided attention. These findings are supported by the results of a recent meta-analysis which included 47 studies examining CPT performance of children with ADHD (Huang-Pollock et al. 2012). Compared to typically developing children, children with ADHD showed decreased CPT performances as indicated by large group differences in the overall test performance (main effects group) but also by small-to-medium TOT effects indicating impairments of sustained attention. A number of studies on adults with ADHD, however, did not confirm the present findings as well as the findings on children with ADHD, as these studies found a preserved task performance over time in adults with ADHD (Epstein et al. 1998, 2001; Tucha et al. 2008). As a major difference to previous research, the present study explored sustained attention by applying multiple tests aiming to measure various aspects of attention. This notion is supported by the only low-to-moderate associations between various measures of sustained attention (Table 2), suggesting that it is indeed relevant and meaningful to consider different components of attention in the assessment of prolonged task performance. Previous studies largely focused on vigilance tasks (e.g., variants of the CPT) which have been criticized by a number of researchers, as (1) vigilance can be assumed to rely on a concept that does hardly represent cognitive demands of daily life activities (Tucha et al. 2009); (2) the validity of CPTs to measure sustained attention is questionable (Swanson et al. 1990), and (3) CPTs have been reported to be of only limited use in differentiating patient groups with different diagnoses (Barkley et al. 1990). It must be noted that the present study did not provide evidence for higher ecological validity of the present tests for the measurement of sustained attention compared to traditional measures such as variants of the CPT. However, the present study aimed to measure various aspects of sustained attention that have high relevance for daily life activities, by selecting and adapting tests on face validity based on task characteristics, and by showing that the applied measures of sustained attention are only weakly to moderately associated. The conclusion of a sustained attention deficit of adults with ADHD drawn on the basis of the present data might thus be of particular clinical importance.

Furthermore, a comprehensive analysis of attention performance requires a differentiation between different types of test variables. In this respect, the present assessment of attention considered the speed of responses (mean reaction time), the accuracy of responses (number of omissions) as well as the intra-individual variability of responses (standard deviation of reaction times). Reaction times of responses represent a general response readiness and arousal, whereas the number of omissions indicates a careless response style or distractibility. Furthermore, the intra-individual variability of responses (e.g., standard deviation of reaction times) is more likely associated with the striking clinical characteristic of frequent lapses of attention seen in patients with ADHD, as it is clinically described by a moment-to-moment variability and inconsistency of performance (Castellanos and Tannock 2002; Castellanos et al. 2005). An increased variability of reaction times was stressed to be an important marker of sustained attention deficits in individuals with ADHD (Castellanos and Tannock 2002; Marchetta et al. 2008). The present study revealed a greater deterioration of attention performance over time in adults with ADHD in all three types of test variables (reaction times, variability of reaction times and number of omissions), although the most pronounced effects were found in number of omissions, suggesting a primary careless response style (e.g., distractibility) of adults with ADHD over time. However, analysis of the numbers of omissions in the different tests for attention revealed low rates of omissions errors in the alertness and selective attention tests, in particular for healthy individuals. These findings might indicate a ceiling effect of healthy individuals. Omission errors observed in the divided attention test might therefore be most informative.

## Implications

In future research, it would be of interest to explore associations between objectively assessed sustained attention deficits of adults with ADHD (as obtained by neuropsychological tests) and subjective complaints of sustained attention (as obtained by self-reports), as this would provide valuable information about the predictive validity of different assessment strategies (Fuermaier et al. 2015). With regard to self-ratings of sustained attention, studies demonstrated that about 84–90 % of patients with ADHD experience deficits with sustaining attention (Downey et al. 1997; Epstein et al. 1998). However, these findings must be interpreted with caution as patients in these studies were asked for the frequency of sustained attention deficits, whereas the patients’ experienced decrement in performance over time (TOT effects) was not measured. As it cannot be assumed that patients with ADHD are familiar with the concept of sustained attention (presence of TOT effects as an indicator of sustained attention deficits), and as a self-rating of the frequency of sustained attention deficits does not measure deterioration of performance over time, conclusions about sustained attention deficits cannot be drawn on the basis of these data. Consequently, it was suggested (Tucha et al. 2009) to perform self-ratings of attention abilities repeatedly during a standardized task in order to examine changes of self-rated cognitive abilities over time (TOT effects). Furthermore, the present and previous data (Tucha et al. 2009) indicate that sustained attention deficits might be present in some but not all aspects of attention, suggesting that self-ratings of sustained attention deficits should distinguish between different types of attention.

Furthermore, the present results of sustained attention deficits in adults with ADHD provide important clinical implications for structuring and scheduling daily activities of patients. It can be hypothesized that individuals with ADHD might benefit from more frequent breaks and by this shorter periods of unbroken continuous task performance. As sustained attention deficits become most obvious with proceeding task duration, breaking cognitive tasks of daily life down into shorter time units might reduce the negative consequences of these deficits. However, it must be considered that other characteristics of individuals with ADHD may argue against this suggestion. For example, even though daily functioning might be improved by subdividing prolonged tasks into shorter units, a normal level of functioning will presumably not be achieved by applying this strategy as adults with ADHD have also been shown to be impaired when cognition was assessed for shorter time periods (Fuermaier et al. 2013b, 2015; Tucha et al. 2006a, 2008). Further, subdividing prolonged tasks into shorter units results in a greater number of units with which individuals have to get engaged with, which may in particular challenge individuals with ADHD because of their difficulties with initiation of tasks and getting into activities (Altgassen et al. 2014; Kerns and Price 2001).

## Limitations

The present study has to be seen in the context of some limitations. Even though data analysis revealed sustained attention deficits of adults with ADHD, a sample size of 29 patients with ADHD limit the generalization of the results to the population of patients with ADHD. This problem is for example reflected in the calculation of significance, since larger samples are required to reveal significance of small effects. In order to address the issue of small sample sizes in hypothesis testing, interpretations of the present data were largely based on effect sizes as they indicate the magnitude of an effect independently of the sample size (Cohen 1988). Interpretation of effect sizes was also favored over statistical significance as multiple testing may have led to alpha-error accumulation.

Furthermore, the group of patients with ADHD consisted of a heterogeneous sample of individuals with regard to subtype (inattentive subtype and combined subtype), comorbidity (e.g., mood and anxiety disorders) and medication status (two patients were currently treated with antidepressant medication). Neuropsychological functions of adults with ADHD have been shown to be associated with various factors, including subtype (Tucha et al. 2008), comorbidity (Seidman 2006), and treatment with antidepressant medication (Amadoboccara et al. 1995), which all might have confounded analysis and conclusions on sustained attention deficits of patients with ADHD in the present study. Unfortunately, the present data set does not allow a reliable analysis of these factors due to small group sizes. For this reason, future research on sustained attention in adults with ADHD should differentiate systematically between patients of various subtypes and presence of comorbidity in order to determine potential factors underlying sustained attention deficits in ADHD. With regard to comorbidity, it must be considered that about 30 % of the patients of the present study had comorbid disorders which may have affected sustained attention functioning and by this may have accounted for the effects. We decided to not exclude patients with ADHD suffering from comorbid psychiatric disorders, because comorbidity is frequent in ADHD (Biederman et al. 1993; Biederman 2005). Consequently, a sample including patients with comorbidity is more representative for the population of ADHD patients than a sample of patients without comorbidity. One study that differentiated between patients with and without comorbidity was conducted by Marchetta et al. (2008). This study, however, did not emphasize the importance of comorbidity as it was concluded that sustained attention deficits were specific to ADHD, regardless of comorbidity.

Moreover, additional information on sustained attention deficits of adults with ADHD might be obtained by applying other tests or analysis techniques. Though the present data were interpreted in terms of deficits in sustained alertness, selective attention, and divided attention, the tasks applied had also demands on inhibitory control and response inhibition. In the alertness task, for example, the target stimulus occurred with 100 % certainty after the fixation cross, requiring the participants to withhold the motor response until the target stimulus appeared. In the tests for selective attention and divided attention, participants were requested to react to target stimuli but to inhibit responses to distractor stimuli (non-targets). Sustained abilities in inhibition is worthwhile studying in adults with ADHD, e.g., by means of TOT effects in Go/No-go tests or Stop-Signal tests. Furthermore, a more fine-grained analysis of sustained attention deficits of adults with ADHD might be achieved by the analysis of intra-individual variability (IIV) of reaction times. Studies analyzing IIV of reaction times showed a greater proportion of extremely long reaction times in both children and adults with ADHD than in typically developing individuals (Gmehlin et al. 2014; Hervey et al. 2006). A study investigating TOT effects of IIV measures of children with ADHD further demonstrated that these abnormally slow reaction times progressively increased with time (Tarantino et al. 2013). Taken together, IIV analyses appear to provide an important mean for the study of sustained attention deficits in ADHD.

As an explanatory model for a more pronounced decrement of task performance over time in individuals with ADHD, it can be suggested that task inefficiency reflects a less optimal energetic state of performance (Van der Meere and Sergeant 1988a, b) which becomes more pronounced with ongoing task duration as novelty of stimuli decreases with time. In line with this argumentation, reaction time performance was shown to vary with length of inter-stimulus intervals (ISI) in both healthy individual and individuals with ADHD (Hervey et al. 2006). It was speculated that ISI may exert its effect by providing more opportunity for distraction or off-task behavior, or possibly by changing the cognitive energy level of individuals (Hervey et al. 2006). A differential investigation of measures according to ISI (long vs. short) might in the present context also be interesting in order to explore the effects of variable foreperiod on anticipatory responses between groups.

Finally, the relevance of sustained attention deficits would be highlighted by demonstrating associations between deficits as shown in neuropsychological assessment and deficits in external measures of impairments. External validation could be achieved by several means, e.g., partner or employers ratings of cognitive functioning, impairment ratings in major life activities (e.g., in the social, educational and occupational environment) or objective measures of functioning in major life activities (such as number of traffic errors, drug use, money management, or numbers of jobs held).
